# Allostery: An Overview of Its History, Concepts, Methods, and Applications

**DOI:** 10.1371/journal.pcbi.1004966

**Published:** 2016-06-02

**Authors:** Jin Liu, Ruth Nussinov

**Affiliations:** 1 Department of Pharmaceutical Sciences, University of North Texas Systems College of Pharmacy, University of North Texas Health Science Center, Fort Worth, Texas, United States of America; 2 Department of Chemistry, Center for Drug Discovery, Design, and Delivery (CD4), Center for Scientific Computation, Southern Methodist University, Dallas, Texas, United States of America; 3 Cancer and Inflammation Program, Leidos Biomedical Research, Inc., Frederick National Laboratory for Cancer Research, National Cancer Institute, Frederick, Maryland, United States of America; 4 Sackler Inst. of Molecular Medicine, Department of Human Genetics and Molecular Medicine, Sackler School of Medicine, Tel Aviv University, Tel Aviv, Israel

## Abstract

The concept of allostery has evolved in the past century. In this Editorial, we briefly overview the history of allostery, from the pre-allostery nomenclature era starting with the Bohr effect (1904) to the birth of allostery by Monod and Jacob (1961). We describe the evolution of the allostery concept, from a conformational change in a two-state model (1965, 1966) to dynamic allostery in the ensemble model (1999); from multi-subunit (1965) proteins to all proteins (2004). We highlight the current available methods to study allostery and their applications in studies of conformational mechanisms, disease, and allosteric drug discovery. We outline the challenges and future directions that we foresee. Altogether, this Editorial narrates the history of this fundamental concept in the life sciences, its significance, methodologies to detect and predict it, and its application in a broad range of living systems.

## Evolution of the Allostery Concept

Allostery, a biological phenomenon commonly referring to regulation at distant sites, has been studied for nearly half a century, even before the word “allostery” was coined. In 1904, Christian Bohr described an interesting biological relationship: one molecule (carbon dioxide) affects the binding affinity of another molecule (oxygen) to a protein (haemoglobin) [1]. This phenomenon—currently known as the “allosteric effect”—was named the “Bohr effect” and studied as cooperative binding of ligands to distinct protein sites. Several equations, such as the Hill [2], Adair [3], Klotz [4], and Pauling [5] equations, have been developed to describe such effects.

The term “allosteric” first appeared in 1961, when Jacques Monod and Francois Jacob [6] used “allosteric inhibition” to describe a mechanism in which “the inhibitor is not a steric analogue of the substrate.” Later in the 1960s, two well-known models were proposed to describe allosteric effects, including the concerted MWC model by Monod, Wyman, and Changeux [7] and the sequential KNF model by Koshland, Nemethy, and Filmer [8]. Since then, for nearly two decades, conformational change was considered as a signature character in the concept of allostery. That was the case until 1984, when Cooper et al. [9] described an allosteric model without conformational change and introduced the term “dynamic allostery,” inserting the entropy contribution into the concept of allostery.

Inspired by the free energy landscape concept, in 1999, the Nussinov group proposed a “conformation selection and population shift” model for molecular recognition [10–12]. This model has been widely used to explain allostery, advancing the concept of allostery from two states (tensed and relaxed) to ensembles of multiple states. Along a different trajectory, in the same year, the Ranganathan group reported energetic connectivity between residues of proteins by examining evolutionarily conserved residues [13] and later identified residues that form allosteric networks for communications between distinct sites of proteins [14]. The “conformation ensembles and population shift” and “allosteric networks” have become two major and complementary points of view of allostery.

In 2004, the Nussinov group further proposed that all proteins are allosteric [15], pushing and promoting a broadened outlook in studies of allostery. In the same year, the US Food and Drug Administration (FDA) approved the first allosteric drug, demonstrating the significance of allostery in therapeutic developments for disease treatment. In 2015, the Nussinov group revisited the “allostery without a conformational change” theory [16] and explained that even if a structural comparison between the active and inactive states does not detect a conformational change, it does not mean that there is no conformational change, and listed likely reasons for this lack of observation. Allostery, as “the second secret of life” [17] proposed by Monod, has been accepted as a key biological phenomenon for understanding biological systems and diseases and established a new paradigm in drug discovery.

**Fig 1:**
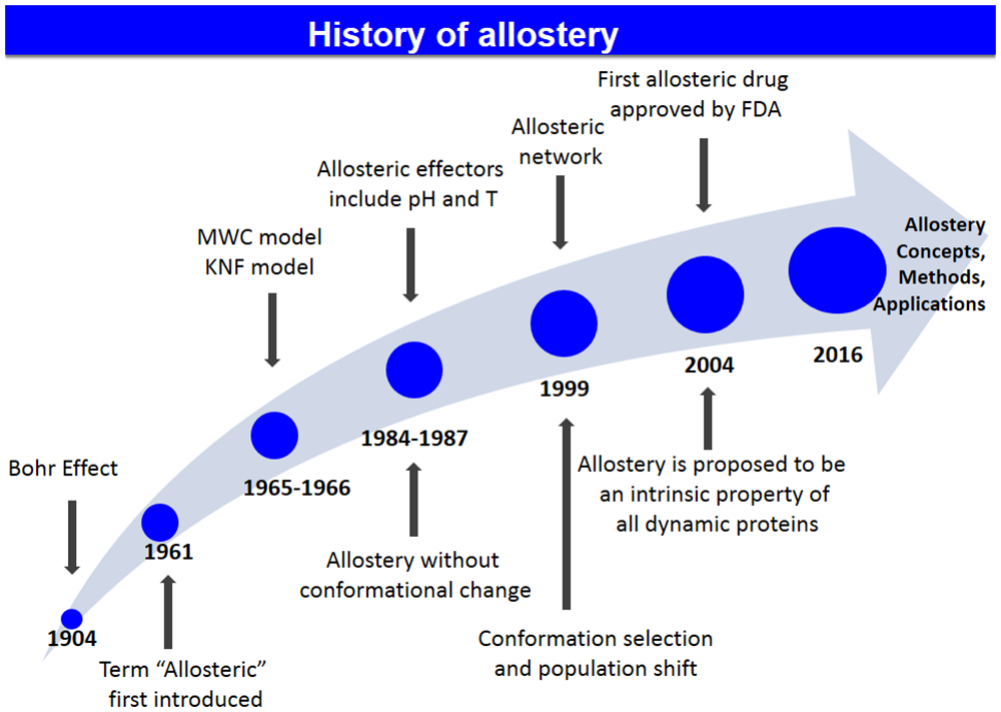
History of allostery.

Recently, the thermodynamics, population shift, and the structural points of view of allostery were unified [18], and allosteric interactions became well established in physiological cell signaling [19], dysfunction in diseases, and drug discoveries [20]. The history of allostery is shown in Fig 1.

In the past three years, more than 1,300 “allostery” papers were published each year, indicating that allostery has become an emerging but still underappreciated field. We hope that this Focus Feature, with articles covering diverse methods and applications in allostery, will help in clarifying this phenomenon and its implications, attracting more attention in the broad scientific community.

## Methods to Study Allostery

Over the five decades of investigation, allostery has been heavily studied by experimental and computational methods. X-ray crystallography is one of the most frequently used experimental approaches. It can provide detailed structural information of the protein before and after perturbation. The difference between “snapshot” structures relating to these two states points to conformational change at the active site upon perturbation at the allosteric site. However, allostery is a dynamical process. The lack of dynamical information on the static crystal structure and possible bias related to conformational change due to crystal packing set limits on X-ray crystallography in studies of allostery. Nuclear magnetic resonance (NMR) spectroscopy methods, on the other hand, capture more “snapshots” on transient conformations that are less populated. In this Focus Feature, NMR methods to study dynamic allostery were thoroughly reviewed by Grutsch et al [21]. Protein conformational changes can be tracked through labels added on the proteins. Well-accepted methods include fluorescence resonance energy transfer (FRET), with the measurement of movement of two fluorophores attached to the protein, and hydrogen–deuterium exchange mass spectrometry (HDXMS), which detects deuterium replacing the hydrogen atom on the protein. A research article by Chandramohan et al. [22] using HDXMS for studying allostery is included in this Focus Feature.

Computational approaches complement experimental methods and provide powerful tools to study allostery, with molecular dynamics simulations providing dynamical details. The large number of snapshots generated from molecular dynamics simulations captures the motion of the proteins, thus providing insights into the population shift of the protein conformational ensemble. Moreover, the prediction power of computational methods enables the identification of allosteric sites, which is essential for allosteric drug discovery. In this collection, a review paper by Hertig et al. [23] discusses the role of molecular dynamics simulations in studies of allosteric mechanisms and allosteric drug discovery. Coarse-grained simulations, as used by Kravats et al. [24] in this collection, has been proven a powerful tool in allosteric investigation of larger biological systems. A new structure-based statistical mechanical model by Guarnera et al. [25] and an improved statistical coupling analysis (SCA) by Rivoire et al. [26], a sequence-based method identifying allosteric network through co-evolved residues, are also included in this collection. The balance between the accuracy of the theoretical models and their computational cost is one of the key factors in developing new methods for protein allostery. With the rapidly decreasing computational cost, we look forward to seeing more accurate computational methods in the near future.

## Applications of Allostery

Allostery regulates biomolecules in a remote action-at-a-distance mode, which is a fundamental requirement for function in cell life. Understanding allostery is essential for in-depth comprehension of a broad range of complex biological systems under physiological conditions and in disease, and will greatly benefit the development of more selective, potent, and effective allosteric drugs.

One of most popular applications of allostery is to understand the mechanism of biological systems. In this Focus Feature, allosteric mechanisms of several proteins, including but not limited to G protein-coupled receptor (GPCR), ClpY ATPase, Hsp90, Aspartate carbamoyltransferase ATCase, NAD-dependent Malic Enzyme (NADME), Catabolite Activator Protein (CAP), Dihydroxyacetone Kinase (DAK), Phosphofructokinase (PFK), and D-3-phosphoglycerate dehydrogenase (PGDH) are discussed, using various methods such as all-atom molecular dynamics simulation, coarse-grained simulations, evolution-based decomposition, HDXMS, and the Structure-Based Statistical Mechanical Model.

Another application of allostery lies in understanding disease and allosteric drug discovery. Fragment-based drug design exploiting the allosteric effects is also discussed in this Focus Feature by Chandramohan et al [22].

## Challenges and Future Directions

Although decades of study and numerous models were proposed for allostery, quantitative description of allosteric communication still remains as one of the biggest challenges. A recent molecular dynamics simulation study by Kalescky et al. [27] reported a surprising phenomenon that increasing protein-local rigidity often leads to the increase of its global configurational entropy, suggesting that the La Châtelier’s principle may be the governing principle behind protein allostery, which opens another venue to understand allostery.

Currently, the rapidly increasing interest in allostery focuses on studies on the single-molecule level. Approaches to depict allostery at the cellular level remain a great challenge. These are critical to advance the concept of allostery from single molecule to cellular regulation. Multi-scale approaches combining experimental methods and computational strategies are needed to span the sizable resolution gap.

We expect that linking the genetic code, which constitutes “the first secret of life,” and allostery, “the second secret of life,” may unlock the still hidden allosteric code in different macromolecular systems and cellular environments and provide new insights into the allosteric enigma. One way is exploiting the evolutionary code to identify allosteric networks; another may be connecting disease, such as cancer, through genome analysis, with allostery to identify the roles that allostery plays. Detailed understanding of allostery in such a framework may lead to new therapeutic developments and disease treatments.

This Focus issue, published in commemoration of the 10th anniversary of PLOS Computational Biology, aims to spotlight allostery. With over 100 years since the publication of the Bohr effect and over 50 years since Monod and Jacob unraveled “allosteric inhibition,” it is fitting to highlight the experimental and computational ways to explore allosteric mysteries, which are fundamental to organismal life. This Focus issue highlights allostery in key proteins, membrane-spanning receptors, and soluble hub proteins, as well as methods to predict allosteric residues, communication pathways, and allosteric sites. It includes single proteins and assemblies, NMR, simulations, and network analysis. We expect it to provide a useful resource to the experimental and computational community. Allostery is an inherent physical phenomenon that nature adopted; breaking its code will benefit the broad community therapeutically and aid in systems design.

## References

[1] Bohr KCH, Krogh A. (1904) Ueber einen in biologischer beziehung wichtigen Einfluss, den die kohlensäurespannung des blutes auf dessen sauerstoffbindung übt. Skandinavisches Archiv Für Physiologie 16: 402–412.

[2] Hill AV (1910) The possible effects of the aggregation of the molecules of hæmoglobin on its dissociation curves. The Journal of Physiology 40.

[3] Adair GS (1925) The hemoglobin system. IV. The oxygen dissociation curve of hemoglobin. J Biol Chem 63: 529–545.

[4] Klotz IM (1946) The application of the law of mass action to binding by proteins; interactions with calcium. Arch Biochem 9: 109–117. 21009581

[5] Pauling L (1935) The Oxygen Equilibrium of Hemoglobin and Its Structural Interpretation. Proc Natl Acad Sci U S A 21: 186–191. 16587956 10.1073/pnas.21.4.186 PMC1076562

[6] Monod J J F.; (1961) General conclusions: telenomic mechanisms in cellular metabolism, growth, and differentiation. Cold Spring Harbor Symp Quant Biol 26: 289–401. 10.1101/sqb.1961.026.01.048 14475415

[7] Monod J, Wyman J, Changeux JP (1965) On the Nature of Allosteric Transitions: A Plausible Model. J Mol Biol 12: 88–118. 14343300 10.1016/s0022-2836(65)80285-6

[8] Koshland DE Jr., Nemethy G, Filmer D (1966) Comparison of experimental binding data and theoretical models in proteins containing subunits. Biochemistry 5: 365–385. 5938952 10.1021/bi00865a047

[9] Cooper A, Dryden DT (1984) Allostery without conformational change. A plausible model. Eur Biophys J 11: 103–109. 6544679 10.1007/BF00276625

[10] Kumar S, Ma B, Tsai CJ, Wolfson H, Nussinov R (1999) Folding funnels and conformational transitions via hinge-bending motions. Cell Biochem Biophys 31: 141–164. 10593256 10.1007/BF02738169

[11] Ma B, Kumar S, Tsai CJ, Nussinov R (1999) Folding funnels and binding mechanisms. Protein Eng 12: 713–720. 10506280 10.1093/protein/12.9.713

[12] Tsai CJ, Kumar S, Ma B, Nussinov R (1999) Folding funnels, binding funnels, and protein function. Protein Sci 8: 1181–1190. 10386868 10.1110/ps.8.6.1181 PMC2144348

[13] Lockless SW, Ranganathan R (1999) Evolutionarily conserved pathways of energetic connectivity in protein families. Science 286: 295–299. 10514373 10.1126/science.286.5438.295

[14] Suel GM, Lockless SW, Wall MA, Ranganathan R (2003) Evolutionarily conserved networks of residues mediate allosteric communication in proteins. Nat Struct Biol 10: 59–69. 12483203 10.1038/nsb881

[15] Gunasekaran K, Ma B, Nussinov R (2004) Is allostery an intrinsic property of all dynamic proteins? Proteins 57: 433–443. 15382234 10.1002/prot.20232

[16] Nussinov R, Tsai CJ (2015) Allostery without a conformational change? Revisiting the paradigm. Curr Opin Struct Biol 30: 17–24. 10.1016/j.sbi.2014.11.005 25500675

[17] Monod J (1977) Chance and Necessity: Essay on the Natural Philosophy of Modern Biology: Penguin Books Ltd.

[18] Tsai CJ, Nussinov R (2014) A unified view of "how allostery works". PLoS Comput Biol 10: e1003394 10.1371/journal.pcbi.1003394 24516370 PMC3916236

[19] Nussinov R, Tsai CJ, Liu J (2014) Principles of allosteric interactions in cell signaling. J Am Chem Soc 136: 17692–17701. 10.1021/ja510028c 25474128 PMC4291754

[20] Nussinov R, Tsai CJ (2013) Allostery in disease and in drug discovery. Cell 153: 293–305. 10.1016/j.cell.2013.03.034 23582321

[21] Grutsch S, Bruschweiler S, Tollinger M (2016) NMR Methods to Study Dynamic Allostery. PLoS Comput Biol 12: e1004620 10.1371/journal.pcbi.1004620 26964042 PMC4786136

[22] Chandramohan A, Krishnamurthy S, Larsson A, Nordlund P, Jannson A and Anand GS (2016) Predicting allosteric effects from orthosteric binding in Hsp90-ligand interactions: Implications for Fragment-based drug design. PLoS Comput Biol 12: e1004840 27253209 10.1371/journal.pcbi.1004840 PMC4890749

[23] Hertig S, Latorraca NR, Dror RO (2016) Revealing atomic-level mechanisms of protein allostery with molecular dynamics simulations. PLoS Comput Biol 12: e1004746 27285999 10.1371/journal.pcbi.1004746 PMC4902200

[24] Kravats AN, Tonddast-Navaei S, Stan G (2016) Coarse-Grained Simulations of Topology-Dependent Mechanisms of Protein Unfolding and Translocation Mediated by ClpY ATPase Nanomachines. PLoS Comput Biol 12: e1004675 10.1371/journal.pcbi.1004675 26734937 PMC4703411

[25] Guarnera E, Berezovsky IN (2016) Structure-Based Statistical Mechanical Model Accounts for the Causality and Energetics of Allosteric Communication. PLoS Comput Biol 12: e1004678 10.1371/journal.pcbi.1004678 26939022 PMC4777440

[26] Rivoire O, Reynolds KA, Ranganathan R (2016) Evolution-Based Functional Decomposition of Proteins. PLoS Comput Biol 12: e1004817 27254668 10.1371/journal.pcbi.1004817 PMC4890866

[27] Kalescky R, Zhou H, Liu J, Tao P (2016) Rigid Residue Scan Simulations Systematically Reveal Residue Entropic Roles in Protein Allostery. PLoS Comput Biol 12: e1004893 10.1371/journal.pcbi.1004893 27115535 PMC4846164

